# The Human Fetus Preferentially Secretes Corticosterone, Rather than Cortisol, in Response to Intra-Partum Stressors

**DOI:** 10.1371/journal.pone.0063684

**Published:** 2013-06-14

**Authors:** Katherine E. Wynne-Edwards, Heather E. Edwards, Trina M. Hancock

**Affiliations:** 1 Department of Comparative Biology and Experimental Medicine, University of Calgary, Calgary, Alberta, Canada; 2 Department of Obstetrics and Gynecology, University of Calgary, Calgary, Alberta, Canada; Fudan University, China

## Abstract

**Context:**

Fetal stress is relevant to newborn outcomes. Corticosterone is rarely quantified in human clinical endocrinology and is found at much lower concentrations than cortisol. However, fetal corticosterone is a candidate hormone as a fetal stress signal.

**Objective:**

Test the hypothesis that preferential fetal corticosterone synthesis occurs in response to fetal intra-partum stress.

**Design:**

Cross-sectional comparison of paired serum corticosteroid concentrations in umbilical artery and vein from 300 women providing consent at admission to a General Hospital Labor and Delivery unit. Pre-term and multiple births were excluded, leaving 265 healthy deliveries.

**Main Outcome Measures:**

Corticosterone and cortisol concentrations determined by LC-MS/MS for umbilical cord venous (V) and arterial (A) samples and used to calculate fetal synthesis (A−V) and proportional fetal synthesis ([A−V]/V). Chart-derived criteria stratified samples by type of delivery, maternal regional analgesia, augmentation of contractions, and clinical rationale for emergent Caesarian delivery.

**Results:**

Cortisol concentrations were higher than corticosterone concentrations; however, the fetus preferentially secretes corticosterone (148% vs 49% proportional increase for cortisol) and differentially secretes corticosterone as fetal stress increases. Fetal corticosterone synthesis is elevated after passage through the birth canal relative to Caesarian deliveries. For vaginal deliveries, augmentation of contractions does not affect corticosteroid concentrations whereas maternal regional analgesia decreases venous (maternal) concentrations and increases fetal synthesis. Fetal corticosterone synthesis is also elevated after C-section indicated by cephalopelvic disproportion after labor, whereas cortisol is not.

**Conclusions:**

The full-term fetus preferentially secretes corticosterone in response to fetal stress during delivery. Fetal corticosterone could serve as a biomarker of fetal stress.

## Introduction

Mechanisms of communication between the human fetus and mother are largely unknown, and are vital to our understanding of parturition, preterm labor and other adverse antenatal events in pregnancy such as preeclampsia and intrauterine growth restriction. Many molecules have been postulated [1]–[3], but only a few have moderate positive predictive value clinically tested in humans [1]–[2]. The role of fetal adrenal steroids as a mechanism for fetal stress signaling to the mother has been problematic to study because fetal cortisol synthesis is difficult to detect against the high background of maternal cortisol. As well, intra-partum cord blood samples have failed to support cortisol as a good biomarker of fetal stress responses *in utero* [4].

As early as 1971, however, significant newborn corticosterone synthesis was established through recovery of injected isotopes of corticosteroid precursors [5]. Comparison of pregnancies with a normal or an anencephalic fetus confirmed that corticosterone of late pregnancy was of fetal origin [6]. Corticosterone remained in fetal peripheral circulation after birth but was slowly replaced by newborn cortisol [7]. An influential paper in 1980 [8], highlighted fetal production of corticosterone in a pregnancy without maternal adrenals, but discussed those data in the context of the potential for fetal corticosterone to serve as a proxy for fetal cortisol, rather than as an independent fetal steroid.

Fetal secretion of corticosterone in response to fetal stress would result in a concentration increase between venous (coming from the placenta towards the fetus) and arterial (moving from the fetus towards the placenta) umbilical circulation. In this context, the current study tested the hypothesis that fetal stress in labor would be signaled through preferential synthesis of corticosterone. After confirmation, by liquid chromatography - tandem mass spectrometry (LC-MS/MS), that the human fetus secretes corticosterone, the study was designed to test the hypothesis that fetal corticosterone synthesis would be positively associated with clinical measures of fetal stress during delivery. Fetal corticosterone synthesis was defined as an increase in corticosterone concentration as blood circulated from the umbilical vein, through the fetus, to the umbilical artery. Specific hypotheses were: 1) Passage through the birth canal after labor (vaginal deliveries) will increase fetal stress, and be reflected in enhanced immediate postnatal fetal corticosterone synthesis, relative to all Caesarian deliveries; 2) Within the group with vaginal delivery, clinical interventions that alter maternal antenatal experiences (maternal regional analgesia or augmentation of contractions with oxytocin analogues) will not alter fetal stress, and therefore will not alter fetal corticosterone synthesis; and 3) With emergency Caesarian delivery, failure to progress in stage 2 of labor, representing the fetal stress of labor with cephalopelvic disproportion, will result in higher fetal corticosterone synthesis than earlier decisions for Caesarian section (i.e., fetal heart rate abnormalities in labor or failure to progress in stage 1 of labor). To assess relative fetal synthesis of corticosterone and cortisol, the same hypotheses were also tested for cortisol.

## Method

### Ethics Statement

This research was granted ethics approval by the University of Calgary Conjoint Health Research Ethics Board (CHREB) as Project E22197. Written consent, on a form approved by the CHREB, was obtained, witnessed, and a copy returned to the participant, from pregnant subjects at the time of admission to labor and delivery. Consent addressed access to labor and delivery chart records and approval for additional umbilical cord blood blood sampling, immediately after the standard clinical practice of obtaining umbilical cord samples for pH and blood gases, to quantify corticosteroid hormones. Placenta and umbilical cord are discarded after the birth, and no access to the infant medical record was needed. Chart data were coded by author HEE, an Obstetrician-Gynecologist, and hormone analyses in the research setting were blind to patient name.

### Subject Pool

Between July 2009 and March 2010, written consent was obtained from 300 pregnant subjects at the time of admission to labor and delivery at the Rockyview General Hospital in Calgary, AB. No attempt was made to assess ethnicity or socioeconomic status. The catchment area for this hospital is predominantly middle class and suburban. Mothers had a mean maternal age of 30.7 years (range 15–42 yr, median 31 yr) and a median parity of 1 (range 0–5). The delivery record provided sex of the baby, birth weight, gestational age and measures of fetal condition at delivery (transfer to the Neonatal Intensive Care Unit (NICU), cord pH, base excess, and Apgar scores at 1, 5 and 10 minutes). After screening the charts to exclude multiple births and pre-term deliveries (≤37 weeks), all of the 265 infants included in the study were considered healthy. Only three infants had APGAR scores ≤6 at 5 minutes and only 15 infants were transiently admitted to the NICU.

### Chart Measures

The following chart-based criteria were used: a) Mode of delivery. The delivery record was used to identify the type of delivery as vaginal, elective Caesarian section without labor, or emergency Caesarian section with history of labor. Successful operative interventions (vacuum or forceps) were coded with Vaginal cases. b) Maternal regional analgesia and/or pharmacological augmentation of contractions. All vaginal deliveries were coded for the presence or absence of maternal regional analgesia and the use or non-use of pharmacological agents to augment contractions (in this unit, Syntocinon®). c) Rationale for emergency Caesarian section. Subjects were divided into three categories of rationale for decision to proceed to emergency Caesarian section: i) Fetal heart rate (FHR) abnormalities in labor (variable decelerations (moderate or severe), late decelerations, decreased variability, and/or tachycardia); ii) Failure to progress/dilate in stage 1 of labor (FTP1; a combination of inadequate contractions and/or early detection of cephalopelvic disproportion); iii) Failure to progress/descend in the pelvis in stage 2 of labor (FTP2; maternal pushing and intense contractions with head compression due to cephalopelvic disproportion).

### Serum Collection

There was no interference with normal post partum protocol which routinely samples umbilical arterial blood for pH and acid-base status. In situations where risk factors for adverse perinatal outcome existed (i.e., emergency Caesarian sections, low 5-minute Apgar score, severe growth restriction, abnormal fetal heart rate tracing, intrapartum fever), both arterial and venous samples were routinely obtained as measures most representative of fetal metabolic condition and arterial acidemia at birth (which quantifies the degree of perinatal asphyxia) [9]. After that routine sampling, but prior to delivery of the placenta, one non-heparinized, ‘red top’, vial of venous and another of arterial whole blood were collected by needle aspiration (16 gauge) for this study. Less than 18% of arterial samples obtained by needle aspiration were expected to be contaminated with venous blood [10]. Samples were immediately refrigerated, and allowed to stand for at least one hour (range 1–12 h, median <4 h), before being centrifuged (4000 G for 5 min). Serum was separated and stored at −20°C until delivery to the research laboratory for hormone analysis.

### Corticosteroid Quantitation

Samples were thawed and a 100 µl serum aliquot was subjected to protein precipitation by the addition of 100 µl of 8.9 mg/ml zinc sulfate containing deuterated internal standards of 10 ng/ml cortisol-d4 and 5 ng/ml corticosterone-d8 (CDN Isotopes Inc, Pointe Claire, QC; D-5280, D-5822). The mixture was vortexed for 30 sec, stored at −20°C for 15 min, and centrifuged at 21000 G for 10 min. A supernatant volume of 150 µl was then transferred to a liquid chromatography vial. Following sample preparation, corticosterone and cortisol concentrations were quantified in a single injection by liquid chromatography- tandem mass spectrometry (LC-MS/MS; [11]–[14]). Specifically, 40 µl of the supernatant was injected onto a 50 mm×2.1 mm, 2.6 µ Kinetex® C18 HPLC column (Phenomenex, Torrance, CA) using an Agilent 1200 LC system with autosampler (Agilent Technologies, Mississauga, ON). The chromatographic separation was performed by gradient elution (15 min) at a flow rate of 0.3 ml/min using Optima grade water containing 0.1% formic acid (mobile phase A) and methanol containing 0.1% formic acid (mobile phase B). The LC system was coupled to an AB Sciex Qtrap® 5500 tandem mass spectrometer (AB SCIEX, Concord, ON) fitted with a Turbo Ion Spray source. The ion spray voltage was set at 5500 V with a source temperature of 350°C. Nitrogen was produced by a high purity nitrogen generator (Parker Balston, Haverhill, MA), and was employed as the curtain, drying and collision gases. Cortisol and corticosterone were monitored in positive ion mode using multiple-reaction monitoring (MRM). MRM transitions used were: Cortisol 363/121; Cortisol-d4 367/121; Corticosterone 347/91; and Corticosterone-d8 355/337. Non-deuterated steroid standards were obtained from Sigma-Aldrich, Oakville, ON (cortisol H4001; corticosterone 27840). LC-MS/MS analyses were conducted over multiple runs in six batches. Cortisol and corticosterone concentrations were determined as area ratios relative to the bio-identical deuterated internal standard (IS) in the same sample and are reported as ng/ml. The intra-assay CV (standard deviation/mean *100) was calculated using the analyte/IS area ratio for six replicates of a low-concentration Quality Control (QC) prepared in charcoal stripped human serum spiked with 50 ng/ml cortisol and 0.5 ng/ml corticosterone, run on a single day. Inter-assay CV could not be calculated by the same method because the IS stock was prepared twice over the study. Instead, the inter-assay CV was calculated from the inferred concentration obtained for six replicates of the identical QC quantified in six different analytical runs on different days. Resulting intra- and inter-assay CV’s were 4.5% and 2.9% for cortisol, and 5.8% and 3.6% for corticosterone. No samples fell outside of the linear range of the calibration curve.

### Analytical Parameters

Fetal corticosteroid synthesis was defined as the concentration difference between the corticosteroid level in the umbilical artery (circulation that has passed the fetus and is returning to the placenta) and the umbilical vein (circulation from the maternal/placental interface towards the fetus). This is represented in the results as [A-V] in ng/ml. In addition, a parameter representing the proportional change in corticosteroid concentration attributable to the fetus ([A-V]/V) was calculated for which negative values represent fetal clearance of corticosteroid, zero represents no change, and values of 1.0 represent a doubling, or 100%, increase in concentration. Analyses also report the absolute concentrations for information. By definition, corticosteroid clearance across the placenta [V-A] was equal in magnitude, but opposite to, fetal synthesis [A-V]. Analytical hypotheses were designed to detect fetal corticosterone synthesis with the prediction that [A-V] corticosterone would be positive ([A-V] >0) and would increase in response to fetal stress. Note that attribution of the concentration change to the materno-placental side of the umbilical circulation, in contrast, would require fetal stress to bypass responses in the fetal adrenal and act, instead, through differential placental clearance of corticosterone and/or decreased maternal corticosterone reaching and crossing the placenta. Thus, parsimoniously, [A-V] >0, and increasing with increasing fetal stress, was interpreted as evidence of fetal corticosterone synthesis in response to fetal stress.

### Statistical Analyses

Distributions for all data were checked for normality, and normality after *log*(*x* +1) transformation. Statistical tests were chosen based on those distributions. Results could not be modeled as a single database, because parameters of interest were not independently distributed (i.e. Caesarian section was always accompanied by maternal analgesia and elective C-section was never preceded by active labor). Instead, subsets of the sample population appropriate for each hypothesis were hierarchically tested. When group variances were unequal (Bartlett test), comparisons between groups used Welch ANOVA for unequal variances with post hoc Dunnett’s Method to compare means against control or Wilcoxon non-parametric comparisons as indicated. All analyses were conducted using JMP version 9.0.2 (SAS Institute, North Carolina) and applied 0.05 as the critical alpha threshold.

## Results

### Both Corticosteroids were Present in Every Sample

Both cortisol and corticosterone were always quantifiable in the umbilical vein (oxygenated blood flowing from the placenta towards the fetus) and umbilical artery (deoxygenated blood flowing from the fetus towards the placenta) samples (**Table 1**). Concentrations of corticosterone and cortisol measured in this study were lower than that reported by radioimmunoassay in previous studies [15]. This is not unexpected since radioimmunoassays detect other steroidal compounds that cross-react with the antibody, in addition to the steroid of interest.

**Table 1:** Median concentration and range for corticosterone and cortisol (ng/ml) in all samples, and samples stratified by mode of delivery.

			Arterial		Venous		
		N	Median†	Range	Median†	Range	Median A-V†
Corticosterone	All	265	6.1	0.7–45	2.2	0.3–13.4	3.1
	Caesarian	53	2.6^a^	0.7–14.9	1.1^a^	0.3–3.9	1.5^a^
	Emergency C	49	4.8^a^	0.8–24.1	1.8^b^	0.5–7.6	2.9^a^
	Vaginal	163	7.6^b^	0.8–44.6	2.9^c^	0.5–13.4	4.2^b^
Cortisol	All	265	80.4	5.9–289	50.8	5.6–269	24.5
	Caesarian	53	35.4^a^	7.7–253.0	23.0^a^	8.0–97.1	12.5^a^
	Emergency C	49	61.9^b^	13.8–210.0	40.7^b^	12.8–224.8	18.0^a^
	Vaginal	163	104.0^c^	5.9–289.0	64.5^c^	5.6–269.0	32.0^b^

† Superscript letters within corticosteroid and column indicate posthoc differences with P<0.05.

In total, only 11/265 subjects had quantitatively similar (−0.25 ng/ml<[A-V] <0.25 mg/ml) or lower (−0.25 ng/ml ≥ [A-V]) cortisol and corticosterone concentrations in the arterial blood samples. A further 22 subjects had a negative or small difference for either cortisol (14/22) or corticosterone (8/22), but not both corticosteroids. Thus, sampling errors mixing venous blood into the arterial sample, or mis-labelling samples, were probably rare (11/265 = 4.2%). Chart records of pH and base excess were not complete (114/265 subjects), limiting the usefulness of pH as a biomarker of venous blood in the arterial samples. No subjects were excluded from subsequent analyses.

After *log*(*x*+1) transformation, cortisol concentration was higher than corticosterone concentration (Paired-t; P<0.0001). Within individual samples, the two corticosteroids had a linear, positive association for absolute concentrations (R^2^ = 0.71; slope = 0.04) and transformed concentrations (R^2^ = 0.65; slope = 0.57). There was no evidence that fetal sex or parity affected the venous concentration of either corticosteroid (all P>0.40).

### Preferential Fetal Corticosterone Synthesis

**Figure 1:**
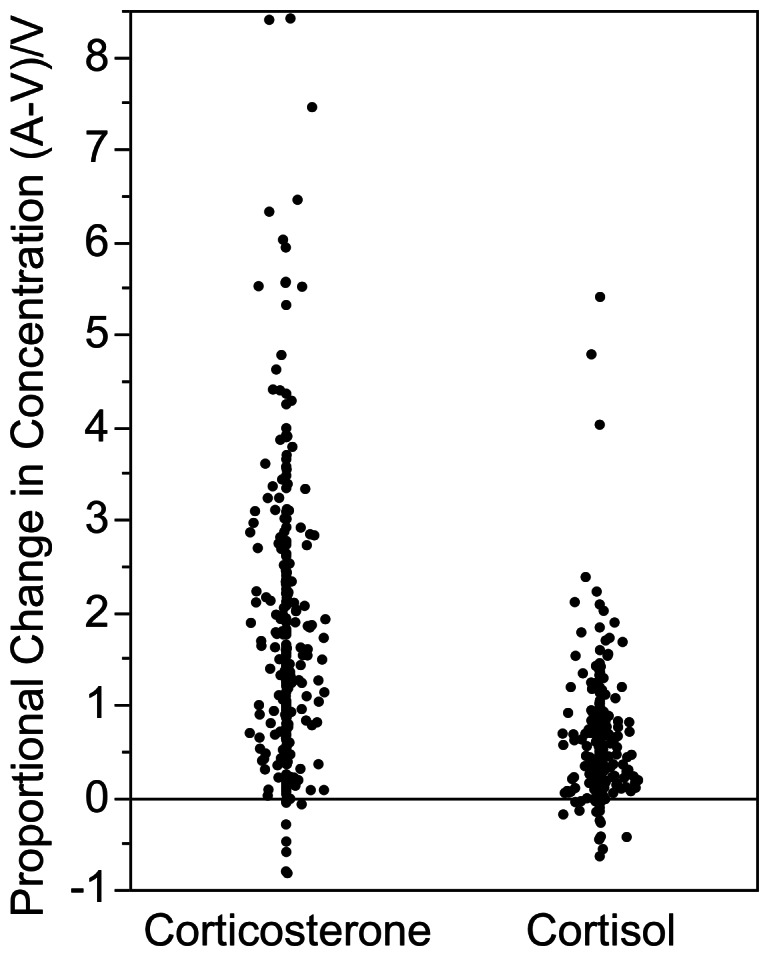
Fetal corticosteroid synthesis. Proportional change in corticosteroid concentrations after passage through the fetus ([A-V]/V) for 265 paired umbilical cord samples. Values near or below zero will include human error accidentally mixing arterial with venous blood during sampling. A value of 1.0 represents a 100% increase, or doubling, of the concentration of corticosteroid from the umbilical vein to the umbilical artery. As the data are intrinsically paired within individuals, statistical comparisons are not shown.

**Figure 2:**
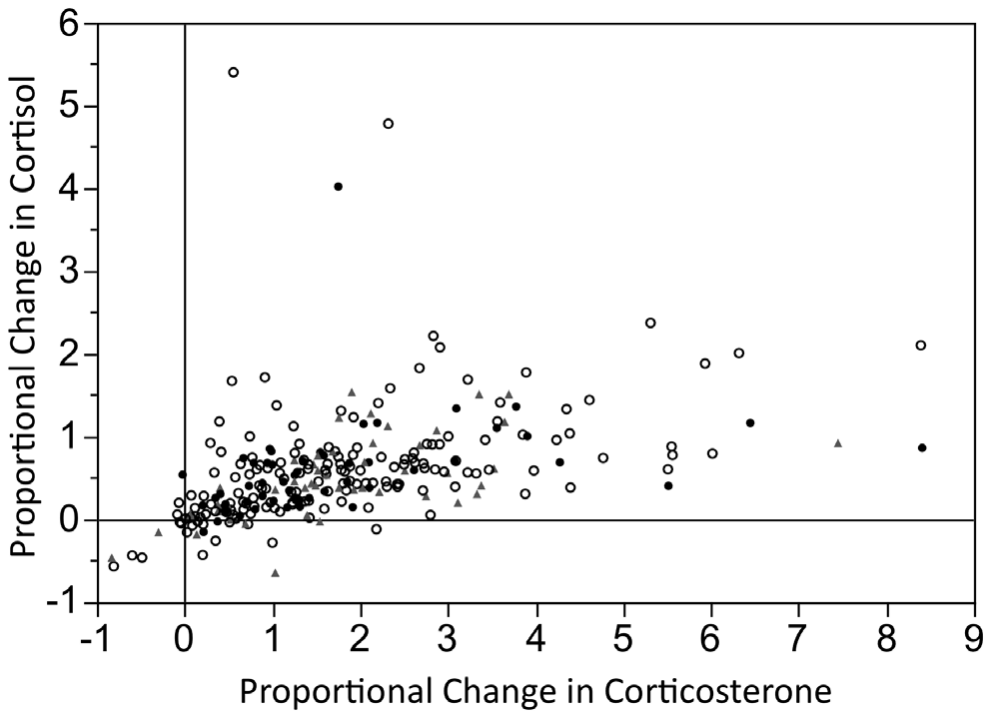
Association between fetal corticosterone and cortisol synthesis by delivery type. Proportional change in corticosteroid concentrations after passage through the fetus ([A-V]/V) for 163 vaginal deliveries (Vag: open circles), 53 scheduled Caesarian sections (C: solid circles), and 49 Emergent C-sections (EC: shaded triangles). Linear positive association, and range of response, are reported in the text.

The median increase in corticosterone concentration [A-V] after passage through the fetus was 3.1 ng/ml with a range from −6.2 to +33.2 ng/ml, representing a median proportional increase of 1.48 ([A-V]/V; range −0.83 to 8.41; **Figure 1**). Cortisol concentration also increased from the venous to the arterial sample with a median increase of 24.5 ng/ml (range −145 to +203 ng/ml), representing a median proportional increase of 0.49 (range −0.64 to +5.40). Thus, the median proportionate increase was three times larger in corticosterone than in cortisol (Paired t = 14.3, df = 264, P<0.0001). As expected, there was an overall positive association between the proportional ([A-V]/V) changes for the two corticosteroids (R^2^ = 0.19; slope = 0.19, P<0.0001; **Figure 2**). Only 3/265 cortisol changes exceed a 250% increase whereas corticosterone changes larger than 250% were common (68/265 = 25.7%). In other words, although the absolute concentrations of cortisol were higher than those of corticosterone, corticosterone continued to increase in subjects where cortisol had reached an apparent maximum. Subsequent analyses tested the hypothesis that fetal corticosterone synthesis would be positively associated with fetal stress.

### Impact of Type of Delivery

**Figure 3:**
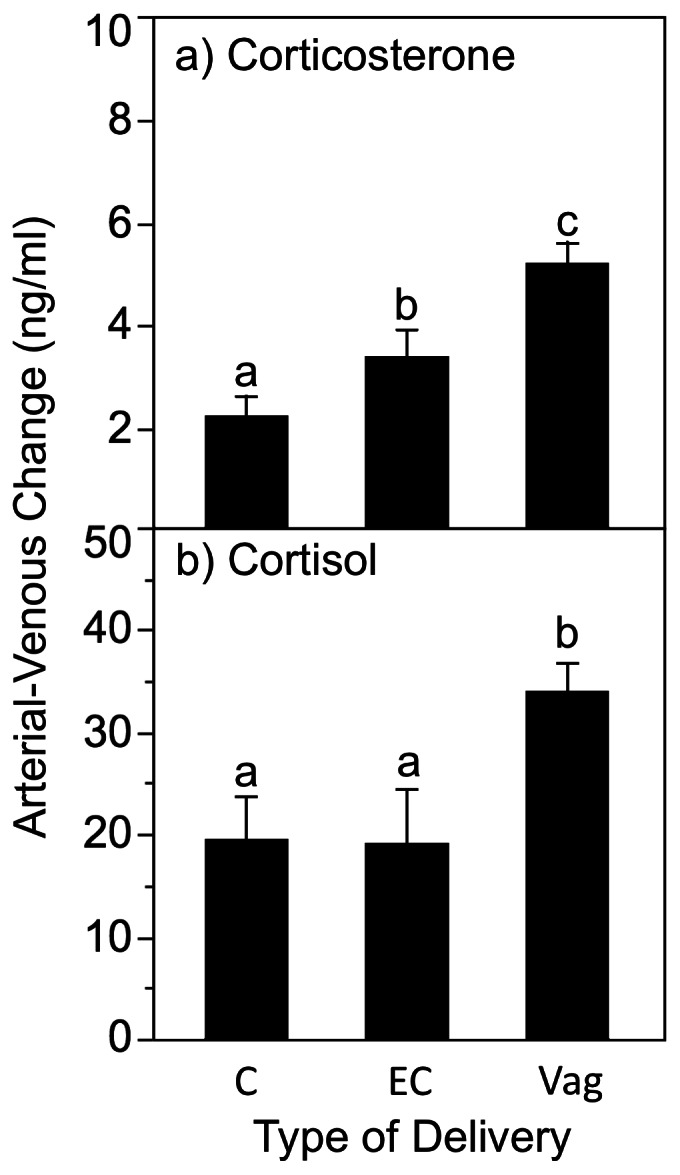
Absolute corticosteroid synthesis differences by delivery type. Absolute increase [A-V] ng/ml in concentration after passage of the fetus for corticosterone (panel a) and cortisol (panel b) across the three types of delivery: scheduled and emergent Caesarian sections (C, EC) and vaginal deliveries (Vag). Letters that differ indicate a significant post-hoc difference between groups following an overall effect of delivery type.

During active labor the *maternal* adrenal gland is highly activated and the placental 11ß- hydroxysteroid dehydrogenase (11ß-HSD) enzyme that converts maternal cortisol to cortisone in the placenta becomes saturated (16). Thus, during active labor 11ß-HSD fails to prevent maternal cortisol from reaching the umbilical venous circulation. For that reason, both corticosterone and cortisol concentrations in the umbilical vein were increased by active labor (Both corticosteroids: Bartlett test for unequal variances P<0.0001, Welch ANOVA P<0.0001). Operative vaginal delivery (forceps or vacuum) did not increase any of the corticosteroid parameters relative to spontaneous vaginal deliveries (all P>0.18). In addition, only 3 of 14 Caesarian section decisions based on failure to progress in Stage 2 (FTP2) followed a failed operative vaginal delivery. Therefore, successful operative deliveries were coded with spontaneous vaginal deliveries, and FTP2 C-sections were coded as Emergency C-sections. Corticosterone and cortisol concentrations in umbilical vein were highest in the vaginal deliveries (Dunnett’s comparison of means against control), intermediate in the emergent C- sections (which also were preceded by active labor), and lowest in elective C-sections without previous labor (**Table 1**). As predicted, fetal corticosterone synthesis [A-V] was elevated (F (2,123.1) = 15.35, P<0.0001) for the vaginal deliveries relative to emergency C-section (P<0.025) and elective C-section (P<0.0001) which also differed (P<0.01; **Figure 3**). Fetal cortisol synthesis was also elevated (F (2,99.4) = 6.22, P<0.005) for the Vaginal deliveries, as compared to both types of Caesarean section delivery (Elective P<0.0001; Emergency P<0.002).

### Impact of Regional Analgesia and/or Augmentation of Contractions in Vaginal Deliveries

**Figure 4:**
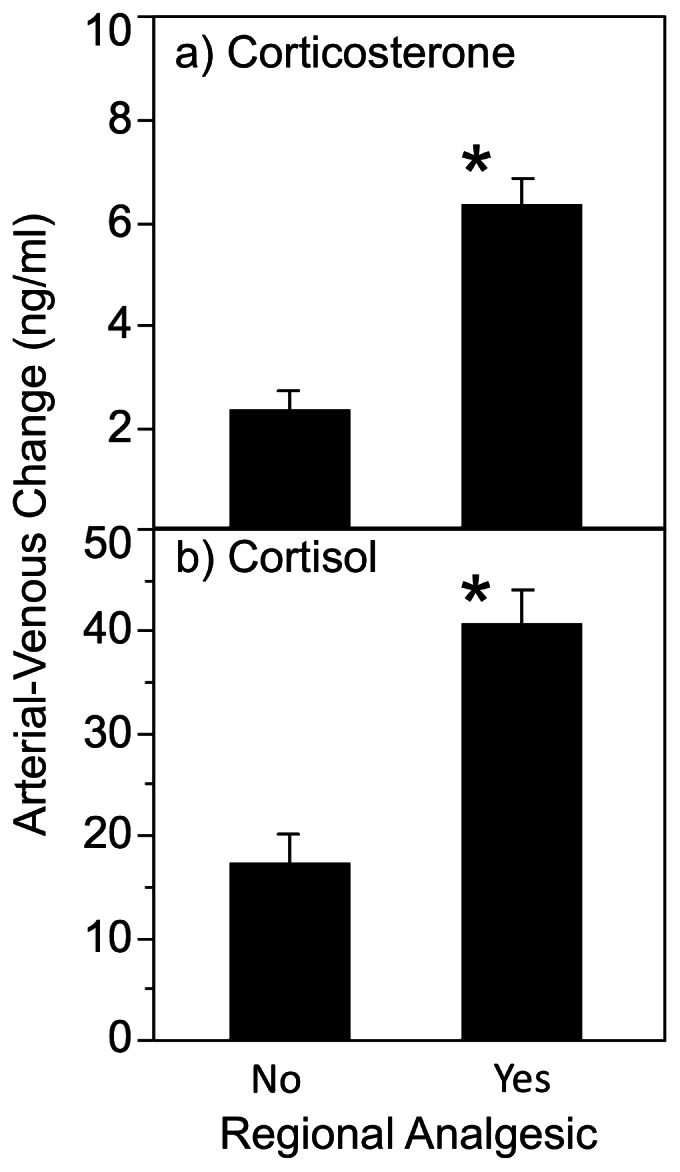
Maternal regional analgesia increases absolute fetal glucocorticoid synthesis. For vaginal deliveries only (Vag), absolute increase [A-V] ng/ml in concentration after passage of the fetus for corticosterone (panel a) and cortisol (panel b) in the absence (N = 46) or presence (N = 117) of regional maternal analgesia (i.e. epidural). The asterisk indicates a significant increase in corticosteroid synthesis in the presence of maternal analgesia.

As seen across the types of delivery, reduction of maternal stress with regional analgesia (followed by vaginal delivery) reduced the concentrations of corticosterone (2.52±0.13 ng/ml versus 4.10±0.40 ng/ml; t unequal variances P<0.0005) and cortisol (56±40 ng/ml versus 86±57 ng/ml; P<0.002) in the umbilical vein flowing from the placenta towards the fetus. However, the hypothesis that fetal corticosteroid synthesis would not be affected by maternal analgesia was not supported. Fetal corticosterone synthesis [A-V] was 3-fold higher in the presence of maternal analgesia (F (1,162) = 11.11; P<0.001: **Figure 4**) and there was no independent effect of maternal augmentation of contractions (P = 0.90) and no interaction between analgesia and augmentation (P = 0.95). Fetal cortisol synthesis also doubled in the presence of maternal analgesia (analgesia F (1,162) = 6.20; P<0.02; augmentation P = 0.71, interaction P = 0.53). Patterns were the same for proportional corticosteroid synthesis. Using the same model, the absolute concentration of cortisol in arterial circulation was not changed by maternal regional analgesia (P = 0.52) although corticosterone concentration was higher in the presence of maternal regional analgesia (P<0.03).

### Rationale for Emergency Caesarean Section

**Figure 5:**
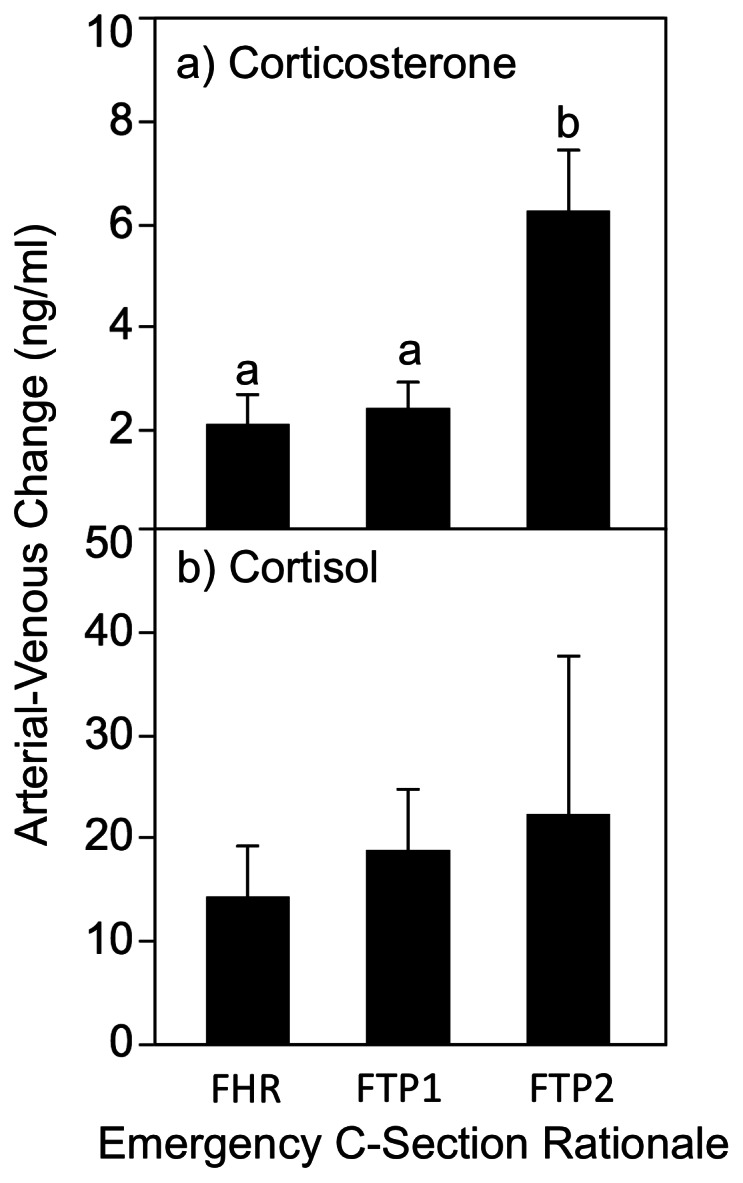
Fetal stress increases fetal corticosterone, but not cortisol, synthesis. For emergent C-sections only (EC: N = 49), absolute increase [A-V] ng/ml in concentration after passage of the fetus for corticosterone (panel a) and cortisol (panel b) in groups stratified by the clinical rationale for the emergent C-section: fetal heart rate abnormalities (FHR: N = 11); Failure to progress in stage 1 (FTP1: N = 19), and failure to progress in Stage 2, representing cephalo-pelvic disproportion detected after a history of labor (FTP2: N = 14). Five EC cases were excluded because the chart record was not clear on the rationale for EC. Letters that differ indicate a significant post-hoc difference between groups, with FTP2 corticosterone higher than FHR and FTP1, following an overall effect of rationale for EC.G.

All infants with fetal heart rate abnormalities had normal cord pH and Apgar scores at 5 minutes, indicating no significant intra-partum asphyxia, or fetal compromise (ie, delivery intervention was early and appropriate). As predicted by the hypothesis that fetal corticosterone synthesis would be positively associated with fetal stress, fetal corticosterone synthesis [A-V] was affected by the rationale for the emergency C-section (F (2, 23.3) = 5.28, P<0.02; **Figure 5**). Corticosterone synthesis was higher for FTP2, which represents elevated fetal stress due to cephalo-pelvic disproportion detected after labor and pushing, than for FHR or FTP1 (P<0.002 and P<0.005). No effect of the rationale for emergency C-section was seen for cortisol synthesis (F (2,24.5) = 0.25, P = 0.78).

## Discussion

**Figure 6:**
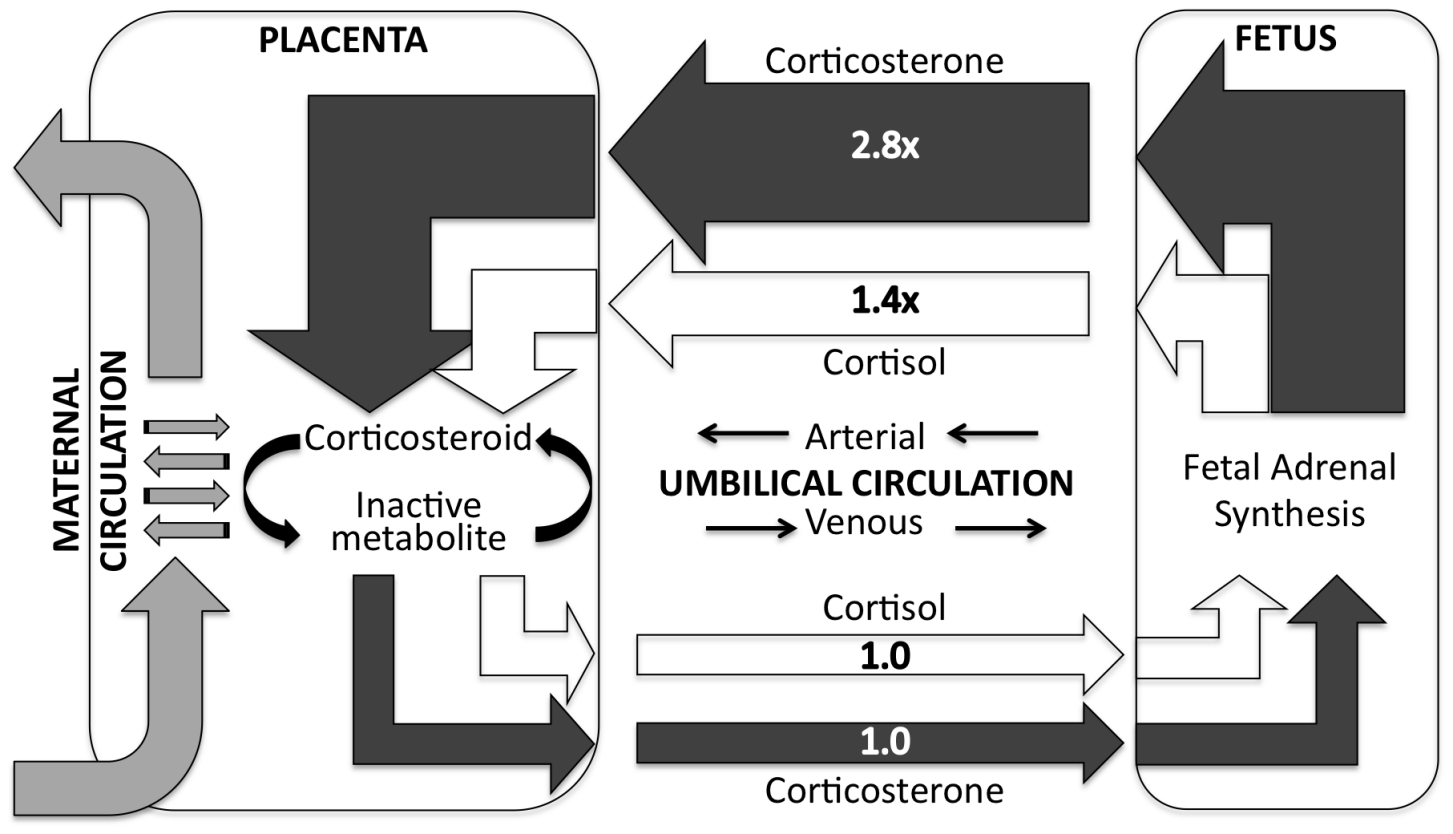
Schematic illustration of changes in median corticosteroid concentrations. Relative concentrations of corticosterone and cortisol in umbilical artery and vein, showing preferential fetal synthesis of corticosterone coupled to placental inactivation of corticosteroids.

Preferential fetal corticosterone synthesis was clearly demonstrated (**Figure 6**). Both cortisol and corticosterone concentrations increased with passage through the fetus, but the overall change in the median cortisol concentration was 1.4× whereas the increase in median corticosterone was 2.8×. A large majority of the matched samples indicated an increase in both corticosteroids from the venous circulation to the arterial circulation. Thus, the fetal adrenal was adding corticosteroids to the arterial circulation, and placental 11ß-HSD was clearing both corticosteroids [16] before the venous circulation returned to the fetus.

In adult humans, cortisol is the dominant corticosteroid produced by the adrenal gland [7], [17]. In contrast, corticosterone is the dominant glucocorticoid produced by the adrenal gland of a large number of other vertebrate species, including reptiles and birds, as well as a range of mammals that includes the laboratory rats and mice typically used as animal models of human stress responses. Corticosterone is synthesized by the adult human adrenal gland, in smaller quantities than cortisol, as a precursor to aldosterone, and is considered less biologically important. Previous studies have measured corticosteroids in the umbilical cord, shortly after delivery [4], [15], [18]–[21], in either the umbilical artery [15], vein [21], or in mixed umbilical cord blood samples [4], [18]. Only two studies to date have compared intra-partum venous and arterial blood samples for both cortisol and corticosterone [19]–[20]. In one, both corticosteroids were elevated in the umbilical vein, but not the umbilical arteries, in women who had premature deliveries (<37 weeks), as compared to women who gave birth to their babies at term [19]. In the second, the A-V difference became positive for cortisol and corticosterone after labor, suggesting fetal corticosteroid synthesis with labor [20]. Thus, developmental changes in fetal glucocorticoid synthesis (cortisol/corticosterone ratio) would be predicted as birth approaches (preferential corticosterone secretion; [15]) and in the early postnatal period (shift towards the adult pattern of preferential cortisol synthesis: [22]–[23]). Normative values for these developmental changes, particularly in the pre-term neonate, are not yet known but could be important for appropriate neonatal diagnosis of adrenal function [24].

Although cortisol concentrations were significantly higher than corticosterone concentrations within paired samples, there is ample evidence that steroids at low concentrations can be biologically potent. For example, the normal range for human estradiol is quantified as pg/ml whereas testosterone, even in women, is found at ng/ml concentrations. Thus, absolute concentration of steroid is a poor predictor of biological potency. Instead, it is clear that cells and tissues readily detect relative concentration changes relative to background concentration (changes in receptor occupancy, etc). In other words, a 10 ng/ml increase against a background of 100 ng/ml is only a 10% increase whereas 10 ng/ml increase against a background of 10 ng/ml is a doubling (100% increase). For that reason, analyses considered the proportional increase in corticosteroid from venous to arterial circulation ([A-V]/V). Preferential proportional fetal corticosterone synthesis occurred irrespective of the concentration arriving at the fetus in the venous circulation, with the proportional increase in corticosteroid at 148% for corticosterone and 49% for cortisol.

Across individuals, the dynamic range for fetal corticosterone synthesis was considerably wider than the equivalent range for cortisol. In fact, the relative rarity of proportional cortisol increases >250% suggested that there might be an intrinsic limit to cortisol increases. This might be a function of negative feedback inhibition from the high background of venous maternal cortisol at birth [4], [20], or even a function of the chemical solubility of cortisol in this flowing circulation, which is largely composed of water. A similar pattern, where the corticosteroid at lower concentrations has a wider dynamic response to sustained, acute stress (capture and handling rather than labor and delivery) has recently been described for diverse wildlife species [14].

To test the hypothesis that fetal corticosterone synthesis intra-partum was positively associated with fetal stress during delivery, patient chart criteria were used, as defined in the study hypotheses, as proxy measures to stratify fetal stress. As expected, the proportional increase in corticosterone was larger than in cortisol, with vaginal delivery following labor highest, and elective Caesarian section lowest. Fetal heart abnormalities resulting in emergent Caesarean sections did not elevate fetal stress, as measured by umbilical corticosterone or cortisol levels, which was not surprising since none of the babies with FHR were compromised based on chart-derived measures. Neither was a fetal stress response detected for women who had an emergent Caesarean section for failure to dilate in the first stage of labor (FTP1). In stark contrast, obstructed labor due to cephalopelvic disproportion (i.e. failure to progress in second stage labor; FTP2) that resulted in an emergency Caesarean section was, as predicted, experienced as stressful by the fetus, with a tripling of corticosterone levels. No comparable response in cortisol was found, which might indicate that the maternal stress response, as measured immediately after delivery by emergency Caesarian section, is similar, irrespective of the clinical rationale for the C-section. Since the proportionate change in corticosterone in the FTP2 group resembles the vaginal delivery group, we infer that head compression into the pelvis (with a successful labor, or an obstructed labor) causes “fetal stress” with a selective tripling of fetal corticosterone biosynthesis and secretion. In future studies, fetal stress during compromised deliveries should also be assessed.

As expected, there was no additional effect of maternal augmentation of contractions on corticosteroid concentrations after progression through the birth canal for vaginal delivery, presumably because stressful passage through the birth canal occurs with and without augmentation. In contrast, fetal corticosteroid synthesis [A-V] was unexpectedly enhanced when the mother had regional analgesia during labor (i.e., epidural). Specifically, the venous concentrations of both corticosteroids arriving at the fetus were reduced by regional analgesia and fetal corticosterone synthesis was increased. Some combination of reduced maternal corticosteroid output, and effective placental 11ß-HSD inactivation, is the probable mechanism for the venous decrease. On the other hand, increased fetal corticosteroid synthesis, in the presence of maternal analgesia, might reflect fetal adrenal release from feedback inhibition previously driven by high venous concentrations. However, this result contrasts with a previous study, with smaller sample size, that found no relation of patient analgesia to venous umbilical cord concentrations of cortisol [21].

During pregnancy, preferential fetal corticosterone synthesis could achieve, through competitive receptor binding at different isoforms of mineralocorticoid and glucocorticoid receptors in the juxtaposed placental arterial and venous vasculature, discrimination between maternal and fetal stress. It is easy to envision the adaptive value to both mother and fetus of being able to detect, and distinguish, fetal from maternal stress [25]. Certainly, there is an emerging literature, involving diverse species including humans, suggesting that developmental and/or early-life stress alters the subsequent stress-phenotype of the individual [26]–[29]. In that context, the possibility that antenatal steroids, routinely provided to mothers at risk for pre-term delivery [29], might alter fetal adrenal corticosterone synthesis [30], also deserves investigation.

## References

[1] Khambay H, Bolt LA, Chandiramani M, De Greeff A, Filmer JE, et al (2012) The Actim Partus test to predict pre-term birth in asymptomatic high-risk women. J Obstet Gynecol 32: 132–134. 22296421 10.3109/01443615.2011.637649

[2] Goldenberg RL, Lams JD, Das A, Mercer BM, Meis PJ, et al (2000) The Preterm Prediction Study: sequential cervical length and fetal fibronectin testing for the prediction of spontaneous preterm birth. National Institute of Child Health and Human Development Maternal-Fetal Medicine Units Network. Am J Obstet Gynecol 182: 636–43. 10739521 10.1067/mob.2000.104212

[3] Honest H, Forbes CA, Duree KH, Norman G, Duffy SB, et al (2009) Screening to prevent spontaneous preterm birth: systematic reviews of accuracy and effectiveness literature with economic modelling. Health Technol Assess 13: 1–627. 10.3310/hta13430 19796569

[4] Gitau R, Menson E, Pickles VM, Fisk NM, Glover V, et al (2001) Umbilical cortisol levels as an indicator of the fetal stress response to assisted vaginal delivery. Eur J Obstet Gynecol Reprod Biol 98: 14–17. 11516793 10.1016/s0301-2115(01)00298-6

[5] Hall CS, Branchaud C, Klein GP, Loras B, Rothman S, et al (1971) Secretion rate and metabolism of the sulphates of cortisol and corticosterone in newborn infants. J Clin Endocrinol Metab 33: 98–104. 5558044 10.1210/jcem-33-1-98

[6] Oakley RE, Cawood ML, Isherwood DM, Heys RF, Shahwan MM (1977) Corticoid biosynthesis by the human fetal adrenal: evidence from measurements in vivo and in vitro. J Steroid Biochem 8: 505–513. 599920 10.1016/0022-4731(77)90253-9

[7] Sippell WG, Becker H, Versmold HT, Bidingmaier F, Knorr D (1978) Longitudinal studies of plasma aldosterone, corticosterone, deoxycorticosterone, progesterone, 17-hydroxyprogesterone cortisol and cortisone determined simultaneously in mother and child at birth and during the early neonatal period. I. Spontaneous delivery. J Clin Endocrinol Metab 46: 971–985. 263476 10.1210/jcem-46-6-971

[8] Fencl MD, Stillman RH, Cohen H, Tulchinsky D (1980) Direct evidence of sudden rise in fetal corticoids late in human gestation. Nature 287(5779): 225–226. 7432458 10.1038/287225a0

[9] Thorp JA, Rushing RS (1999) Umbilical cord blood gas analysis. Obstet Gynecol Clin North Am 26(4): 695–709. 10587963 10.1016/s0889-8545(05)70107-8

[10] Westgate J, Garibaldi J, Greene KR (1994) Umbilical cord blood gas analysis at delivery: a time for quality data. Br J Obstet Gynaecol 101(12): 1054–63. 7826958 10.1111/j.1471-0528.1994.tb13581.x

[11] Soldin SJ, Soldin OP (2009) Steroid hormone analysis by tandem mass spectrometry. Clin Chem 55: 1061–1066. 19325015 10.1373/clinchem.2007.100008 PMC3634331

[12] Guo T, Taylor RL, Singh RH, Soldin SJ (2006) Simultaneous determination of 12 steroids by isotope dilution liquid chromatography-photospray ionization tandem mass spectrometry. Clin Chim Acta 372: 76–82. 16707118 10.1016/j.cca.2006.03.034

[13] Koren L, Ng ESM, Soma KK, Wynne-Edwards KE (2012) Sample preparation and liquid chromatography-tandem mass spectrometry for multiple steroids in mammalian and avian circulation. PLoS One 7: e32496. 22384262 10.1371/journal.pone.0032496 PMC3288106

[14] Koren L, Whiteside D, Fahlman A, Ruckstuhl K, Kutz S, et al (2012) Cortisol and corticosterone independence in cortisol-dominant wildlife. Gen Comp Endocrinol 177: 113–119. 22449618 10.1016/j.ygcen.2012.02.020

[15] Dörr HG, Versmold HT, Bidlingmaier F, Sippell WG (1989) Adrenocortical steroids in small-for-gestational-age term infants during the early neonatal period. Pediatr Res 25(2): 115–118. 2919124 10.1203/00006450-198902000-00001

[16] Martinerie L, Pussard E, Meduri G, Delezoide A-L, Boileau P, et al (2012) Lack of Renal 11 Beta-Hydroxysteroid Dehydrogenase Type 2 at Birth, a Targeted Temporal Window for Neonatal Glucocorticoid Action in Human and Mice. PLoS ONE 7: e31949 doi:10.1371/journal.pone.0031949. 22359645 10.1371/journal.pone.0031949 PMC3281096

[17] Pepe GJ, Albrecht ED (1995) Actions of placental and fetal adrenal steroid hormones in primate pregnancy. Endocr Rev 16: 608–648. 8529574 10.1210/edrv-16-5-608

[18] Homoki J, Teller WM, Tschurtz D, Fazekas ATA (1975) The concentrations of total cortisol and corticosterone in mixed cord plasma. Acta Paediatr Scand 64: 587–591. 1155078 10.1111/j.1651-2227.1975.tb03887.x

[19] Ostra A, Toropila M, Pacin J, Kacmajrik J (1996) Adrenal cortex hormones in the mother and fetus in premature labor. Ceska Gynekol 61: 86–90. 8689006

[20] Sippell WG, Dorr HG, Becker H, Bidlingmaier F, Mickan H, et al (1979) Simultaneous determination of seven unconjugated steroids in maternal venous and umbilical arterial and venous serum in elective and emergency cesarean section at term. Am J Obstet Gynecol 135: 530–42. 484653 10.1016/0002-9378(79)90445-9

[21] Jaskot B, CzeszyAska MB, KonefaA H, Pastuszka J (2011) Method of analgesia for labor in relation to newborn condition, cord blood cortisol and interleukin-6 levels. Ginekol Pol 82: 767–74. 22379941

[22] Dörr HG, Sippell WG, Versmold HT, Bidlingmaier F, Knorr D (1988) Plasma mineralocorticoids, glucocorticoids, and progestins in premature infants: longitudinal study during the first week of life. Pediatr Res 23: 525–529. 3387174 10.1203/00006450-198805000-00018

[23] Sippell WG, Dörr HG, Bidlingmaier F, Knorr D (1980) Plasma levels of aldosterone, corticosterone, 11-deoxycorticosterone, progesterone, 17-hydroxyprogesterone, cortisol and cortisone during infancy and childhood. Pediatr Res 14: 39–46. 7360520 10.1203/00006450-198001000-00010

[24] Aucott SW (2012) The challenge of defining relative adrenal insufficiency. J Perinatol 32: 397–398. 22643290 10.1038/jp.2012.21

[25] Love OP, Chin EH, Wynne-Edwards KE, Williams TD (2005) Stress hormones: a link between maternal condition and sex-biased reproductive investment. Am Nat 166: 751–766. 16475090 10.1086/497440

[26] Graf AV, Dunaeva TYu, Maklakova AS, Maslova MV, Sokolova NA (2012) Transgenerational effects of prenatal stress of different etiology. Biol Bull 39: 448–457. 23136741

[27] Pawluski JL, Brain UM, Underhill CM, Hammond GL, Oberlander TF (2012) Prenatal SSRI exposure alters neonatal corticosteroid binding globulin, infant cortisol levels, and emerging HPA function, Psychoneuroendocrinology. 37: 1019–1028. 10.1016/j.psyneuen.2011.11.011 22177580

[28] Harkness KL, Stewart JG, Wynne-Edwards KE (2011) Cortisol reactivity to social stress in adolescents: Role of depression severity and child maltreatment Psychoneuroendocrinology. 36: 173–81 doi: 10.1016/j.yhbeh.2011.01.004. 10.1016/j.psyneuen.2010.07.006 20688438

[29] ACOG Committee on Obstetric Practice (2011) ACOG Committee Opinion No 475: antenatal corticosteroid therapy for fetal maturation. Obstet Gynecol 117(2 pt 1): 422–424. 10.1097/AOG.0b013e31820eee00 21252775

[30] Keelan JA, Mattes E, Tan H, Dinan A, Newnham JP, et al (2012) Androgen concentrations in umbilical cord blood and their association with maternal, fetal and obstetric factors. PLoS ONE 7: e42827 doi:10.1371/journal.pone.0042827. 22916165 10.1371/journal.pone.0042827 PMC3423422

